# Association of diabetes and diabetes treatment with incidence of breast cancer

**DOI:** 10.1007/s00592-015-0756-6

**Published:** 2015-04-29

**Authors:** Esther García-Esquinas, Elisabeth Guinó, Gemma Castaño-Vinyals, Beatriz Pérez-Gómez, Javier Llorca, Jone M. Altzibar, Rosana Peiró-Pérez, Vicente Martín, Concepción Moreno-Iribas, Adonina Tardón, Francisco Javier Caballero, Montse Puig-Vives, Marcela Guevara, Tania Fernández Villa, Dolores Salas, Pilar Amiano, Trinidad Dierssen-Sotos, Roberto Pastor-Barriuso, María Sala, Manolis Kogevinas, Nuria Aragonés, Víctor Moreno, Marina Pollán

**Affiliations:** Environmental and Cancer Epidemiology Unit, National Centre for Epidemiology, Carlos III Institute of Health (Instituto de Salud Carlos III–ISCIII), Avda. Monforte de Lemos, 5, 28029 Madrid, Spain; Department of Preventive Medicine and Public Health, School of Medicine, Universidad Autónoma de Madrid, Madrid, Spain; IdiPAZ, Madrid, Spain; Consortium for Biomedical Research in Epidemiology and Public Health (CIBER en Epidemiología y Salud Pública–CIBERESP), Madrid, Spain; Unit of Biomarkers and Susceptibility, Catalan Institute of Oncology (ICO-IDIBELL), Barcelona, Spain; Centre for Research in Environmental Epidemiology (CREAL), Barcelona, Spain; IMIM (Hospital del Mar Medical Research Insititute), Barcelona, Spain; Universitat Pompeu Fabra (UPF), Barcelona, Spain; Institute of Health Research “Puerta de Hierro”, IDIPHIM, Madrid, Spain; Division of Epidemiology and Computational Biology, School of Medicine, University of Cantabria, IDIVAL, Santander, Spain; Public Health Divission of Gipuzkoa, BioDonostia Research Institute, San Sebastián, Spain; Dirección General de Salud Pública, Fundación para el fomento de la investigación sanitaria y biomédica de la Comunidad Valenciana, FISABIO-Salud Pública, Barcelona, Spain; Grupo de Investigación en Interacción Gen-Ambiente-Salud, Department of Preventive Medicine and Public Health, University of Leon, Leon, Spain; Public Health Institute of Navarra, Pamplona, Spain; Red de Investigación en Servicios de Salud en Enfermedades Crónicas (REDISSEC), Pamplona, Spain; Instituto Universitario de Oncología, University of Oviedo, Asturias, Spain; Hospital Infanta Elena de Huelva, Huelva, Spain; Centro de Investigación en Salud y Medio Ambiente (CYSMA), Universidad de Huelva, Huelva, Spain; Epidemiology Unit and Girona Cancer Registry (UERCG), Oncology Coordination Plan (PDO), Department of Health, Autonomous Government of Catalonia, Barcelona, Spain; Department of Epidemiology and Evaluation, IMIM (Hospital del Mar Medical Research Institute), Barcelona, Spain; University of Barcelona, Barcelona, Spain; School of Public Health, Athens, Greece

**Keywords:** Diabetes Mellitus, Breast cancer, Triple-negative breast neoplasms, Metformin, Insulin

## Abstract

**Aims:**

The aim of this study was to evaluate the association of diabetes and diabetes treatment with risk of postmenopausal breast cancer.

**Methods:**

Histologically confirmed incident cases of postmenopausal breast (*N* = 916) cancer were recruited from 23 Spanish public hospitals. Population-based controls (*N* = 1094) were randomly selected from primary care center lists within the catchment areas of the participant hospitals. ORs (95 % CI) were estimated using mixed-effects logistic regression models, using the recruitment center as a random effect term. Breast tumors were classified into hormone receptor positive (ER+ or PR+), HER2+ and triple negative (TN).

**Results:**

Diabetes was not associated with the overall risk of breast cancer (OR 1.09; 95 % CI 0.82–1.45), and it was only linked to the risk of developing TN tumors: Among 91 women with TN tumors, 18.7 % were diabetic, while the corresponding figure among controls was 9.9 % (OR 2.25; 95 % CI 1.22–4.15). Regarding treatment, results showed that insulin use was more prevalent among diabetic cases (2.5 %) as compared to diabetic controls (0.7 %); OR 2.98; 95 % CI 1.26–7.01. They also showed that, among diabetics, the risk of developing HR+/HER2− tumors decreased with longer metformin use (OR_per year_ 0.89; 95 % CI 0.81–0.99; based on 24 cases and 43 controls).

**Conclusion:**

This study reinforces the need to correctly classify breast cancers when studying their association with diabetes. Given the low survival rates in women diagnosed with TN breast tumors and the potential impact of diabetes control on breast cancer prevention, more studies are needed to better characterize this association.

## Introduction

Diabetes mellitus and cancer are two leading causes of death and disability worldwide. In Europe alone, by 2011 there were around 52.6 million people with diabetes, and 10 % of deaths in adults were attributed to this disease [1]. In this same continent, an estimated 3.4 million cancer cases and 1.75 million cancer deaths occurred during 2012. Breast, colorectal, prostate and lung tumors represented more than half of the overall burden of cancer [2].

Substantial evidence supports that type 2 diabetes is a risk factor for the development of numerous types of cancer, including those of the pancreas, liver, stomach, colorectum, kidney, bladder, postmenopausal breast and endometrium [3]. Still, a number of important questions remain unanswered. First, it is unclear whether this association can be attributed to the existence of common risk factors for both diseases (i.e., age, obesity, lack of physical activity), or to the direct effect of insulin resistance and its compensatory hyperinsulinemia and hyperglycemia. Second, the latency period from diabetes exposure to cancer risk is unknown. Because changes in glucose concentrations, insulin sensitivity or insulin secretion can precede diagnosis of diabetes to up to 6 years [4], the increased risk of cancer could also predate clinical diagnosis of diabetes [5]. Finally, anti-diabetes medication may also modulate the risk of cancer, and further research is needed to disentangle the effects of diabetes from those derived from its treatment. This is even more difficult if we take into account that most diabetic patients are treated with more than one glucose-lowering drug at the same time and that treatment schemes change over the course of the disease according to its severity [6].

There is epidemiological evidence showing that the association between breast cancer and its risk factors vary according to the expression of tumor receptors. However, few previous reports have explored the possible role of diabetes on specific breast cancer subtypes [7]. The objective of this report is to evaluate the association between diabetes and diabetes treatment with incidence of postmenopausal breast cancers, overall and by specific subtypes.

## Materials and methods

MCC is a population-based multicase–control study in Spain. Incidence cases of histologically confirmed breast tumors were recruited from 23 Spanish public hospitals from 2008 to 2013. Inclusion criteria required that cases had lived for at least 6 months in the same study area and were 20–85 years of age. Age- and region-matched population controls were randomly selected from primary care center lists. Response rates were 71 % for breast cancer and 72 % for controls, with no differences in the main socio-demographic variables among those who participated and those who refused to participate. All participants who agreed to participate signed an informed consent, and the study was formally approved by the corresponding ethics committee of each area. The MCC-Spain study also followed the Declaration of Helsinki and the Spanish Personal Data Protection Act of 1999.

Both cases and controls were interviewed by trained personnel, collecting data on socio-demographic factors, health behaviors, gynecologic/obstetric history, preexisting medical conditions, treatments received and family history of cancer. Waist and hip circumferences were measured at the time of the interview.

In the present study, we initially included all cases of postmenopausal breast cancer (*N* = 1018) and their matched controls (*N* = 1243). We then excluded women with lack of information on diabetes status (*N* = 43 women) and, in order to reduce the probability of including type 1 diabetic individuals as exposed, women who had been diagnosed of diabetes before the age of 45 (*N* = 19 women). To allow for a minimum latency period and to avoid that the clinical conditions that lead to diabetes and cancer diagnosis could overlap, we also excluded women who had been diagnosed of diabetes ≤1 year before the diagnosis of cancer (*N* = 12 women;). Finally, to obtain effect estimators adjusted by BMI, we excluded 177 women with missing values, leading to a final sample of 916 cases/1094 controls. In a sensitivity analysis, we included these participants and imputed their BMI to check the consistency of our results (data not shown).

### Variables definition

Time since diagnosis was computed as the age at interview minus the age at fist diagnosis of diabetes. To allow for a minimum latency period, all potential confounders that could be modified by the disease (tobacco and alcohol consumption, energy intake, physical exercise) were censored to 1 year prior to the interview.

Self-reported diabetic drugs were classified according to the Anatomical Therapeutic Chemical Classification System of the World Health Organization. Because the number of participants per subgroup was small, only two main categories are considered for this report: A10A (insulin and analogs) and A10B (blood glucose-lowering drugs, excluding insulin). In accordance with this, diabetic participants were classified into three groups if they had ever received this treatment for at least 1 year: (1) conservative therapy, (2) treatment with blood glucose-lowering drugs and (3) treatment with insulin (±anti-diabetic agents).

### Tumor classification

Trained personnel reviewed all pathologic records and registered information regarding histological type and receptor status in breast cancer cases. Breast tumors were divided into groups according to the presence/absence of the estrogen receptor (ER), progesterone receptor (PR) or the human epidermal growth factor receptor (HER2) as follows: (1) hormone receptor-positive tumors (ER+ or PR+ with HER2−); (2) HER2+ tumors (independent of ER or PR) and (3) triple-negative (TN) tumors (ER−, PR− and HER2−).

### Statistical analyses

Descriptive statistics of participant characteristics were calculated for both cases and controls by diabetes status (Table 1). To evaluate the association between diabetic status and cancer risk, we fitted multivariate logistic mixed models, including the study region as a random effect term, and adjusted for age, educational level, BMI, age of menarche, age at first birth, existence of previous biopsies and family history of the studied cancer. To study whether diabetes treatment could be associated with cancer incidence, we followed two strategies. First, we evaluated the risk of cancer associated with different treatment regimens (conservative, oral medication, insulin ± oral medication) using the non-diabetic population as the reference category. Then, we quantified the association between duration of use of specific anti-diabetic drugs and cancer risk in the diabetic subgroup.

**Table 1:** Main characteristics of the female population by diabetic and cancer status

Characteristics	Cases		p-val_1_^a^	Controls		p-val_1_^a^	p-val_2_^b^
	No DM (*N* = 835)	DM (*N* = 81)		No DM (*N* = 995)	DM (*N* = 99)		
Age	62.0 (8.7)	68.9 (8.0)	<0.01	64.1 (9.14)	71.7 (8.4)	<0.01	<0.01
Study level							
No studies	168 (20.1)	33 (40.7)		192 (19.3)	41 (41.4)		
<High school	319 (38.2)	27 (33.3)		357 (35.9)	32 (32.3)		
High school	235 (28.1)	15 (18.6)		283 (28.4)	16 (16.2)		
>High school	113 (13.5)	6 (7.4)	<0.01	163 (16.4)	10 (10.1)	<0.01	0.32
BMI (kg/m^2^)							
<25	316 (37.8)	14 (17.3)		462 (46.4)	19 (19.2)		
25–30	338 (40.5)	29 (35.8)		339 (34.1)	38 (38.4)		
>30	181 (21.7)	38 (46.9)	<0.01	194 (19.5)	42 (42.4)	<0.01	<0.01
Age menarche							
<12 years	171 (20.5)	13 (16.1)		213 (21.4)	23 (23.2)		
12–13 years	378 (45.2)	39 (48.1)		445 (44.7)	33 (33.4)		
>13 years	286 (34.3)	29 (35.8)	0.69	337 (33.9)	43 (43.4)	0.09	0.17
Age first birth	27.0 (26.3)	27.0 (4.8)	0.21	26.0 (26.4)	27 (26.4)	0.97	0.75
Family history BC							
No	721 (86.4)	72 (88.9)		905 (91.0)	83 (83.8)		
Yes	114 (13.7)	9 (11.1)	0.52	90 (9.1)	16 (16.2)	0.02	<0.01
Previous biopsies							
No	742 (88.9)	76 (93.8)		979 (98.4)	98 (99.0)		
Yes	93 (11.1)	5 (6.2)	0.17	16 (1.6)	1 (1.0)	0.65	<0.01
Screening behavior (mammogram last 5 years)							
No				91 (8.8)	17 (15.9)		
Yes				949 (91.3)	90 (84.1)	0.02	<0.01
DM characteristics							
Duration	–	8.73 (7.9)		–	8.6 (7.1)		0.95
Treatment							
Conservative	–	14 (17.3)		–	21 (21.2)		
Drugs	–	67 (82.7)		–	78 (78.8)		0.51
*Tumor characteristics*							
Bilateral							
Yes	798 (97.6)	79 (97.5)		–	–		
No	20 (2.4)	2 (2.5)	0.99	–	–		–
Histologic subtype							
Ductal	608 (85.0)	64 (87.7)		–	–		
Lobular	53 (7.4)	4 (5.5)		–	–		
Other	54 (7.6)	5 (6.9)	0.80	–	–		–
*Subtypes							
RH+, HER2−	529 (73.1)	51 (66.2)		–	–		
HER2+	121 (16.7)	9 (11.7)		–	–		
Triple−	74 (10.2)	17 (22.1)	0.01	–	–		–

Data are number of participants (percentage) for categorical variables or means (SD) for continuous variables

*DM* diabetes mellitus

^a^p-val_1_: *p* value from Chi-square or ANOVA test for differences in the distribution of the studied variables by diabetic status; ^b^ p-val_2_: *p* value from Chi-square or ANOVA test for differences in the distribution of the studied variables by case/control status

* Numbers in tables differ because not all tumors could be classified according to intrinsic subtypes

To explore whether the effects of diabetes, diabetes treatment or diabetes duration could differ by cancer subtype, multinomial logistic models were fitted, considering in each case the aforementioned subtypes of breast cancer. Heterogeneity of effects was tested using a Wald test comparing the coefficients obtained for the different subtypes.

As sensitivity analyses, we first adjusted all models for waist-to-hip circumference instead of BMI. Second, we further adjusted the models for tobacco and alcohol consumption, energy intake and physical exercise when this information was available (*N* = 1732). Third, we explored the results of including participants with missing values in BMI (*N* = 177) after performing multiple imputation on this variable. Fourth, we further adjusted models on diabetes treatment by diabetes duration. Finally, we evaluated whether the effect of diabetes varied across categories of BMI by introducing an interaction term between the independent variable and BMI (non-obese vs. obese). All sensitivity analyses gave similar results (data not shown in tables).

## Results

Table 1 shows the main characteristics of the study participants. Compared to their controls, cases were slightly younger and had greater BMI values. Diabetic women were older, had lower education levels, higher prevalence of obesity and were more likely to be diagnosed with TN tumors than those without diabetes.

Table 2 shows the results for the association between diabetes status, diabetes management and breast cancer risk, overall and by subtypes. After multivariate adjustment, diabetic women showed no overall increased risk of breast cancer when compared to non-diabetics (OR 1.09; 95 % CI 0.82–1.45). However, significant heterogeneity of the effect was observed by intrinsic subtypes (p_heterogeneity_ < 0.01), with a positive association encountered for TN tumors (OR 2.25; 95 % CI 1.22–4.15). Compared to non-diabetics, diabetic women under conservative management, as well as those under treatment with oral hypoglycemic agents, showed no overall increased risk of breast cancer, although, again, the results suggested a heterogeneous effect by tumor subtype. Conservative management was associated with a nonsignificant risk of HER2 tumors, while treatment with oral hypoglycemic agents alone showed a positive link with TN breast cancer. Diabetes treatment with insulin was associated with an overall increased risk of breast cancer (OR 2.98; 95 % CI 1.26–7.01).

**Table 2:** OR (95 CI) for the association between diabetes, diabetes management and breast cancer in MCC-Spain, overall and by tumor subtype

Exposure variables	Controls	Overall		HR +/HER2−		HER2+		TN		p-het
		Cases	OR (95 %CI)	Cases	OR (95 %CI)	Cases	OR (95 %CI)	Cases	OR (95 %CI)	
*Overall population*										
Diabetes										
Non-diabetic	995	836	1.00	530	1.00	121	1.00	74	1.00	
Diabetic	99	80	1.09 (0.82–1.45)	51	0.94 (0.68–1.29)	10	0.83 (0.38–1.85)	17	**2.25 (1.22**–**4.15)**	**<0.01**
Diabetes management										
Non-diabetic	995	724	1.00	529	1.00	121	1.00	74	1.00	
Conservative management	21	14	0.92 (0.45–1.88)	6	0.49 (0.18–1.32)	5	2.00 (0.99–4.71)	1	*	**<0.01**
Treatment with oral hypoglycemic agents	70	46	0.83 (0.55–1.25)	32	0.85 (0.58–1.25)	2	*	11	**2.13 (1.08**–**4.23)**	**<0.01**
Treatment with insulin (± oral hypoglycemic agents)	8	20	**2.98 (1.26–7.01)**	13	2.94 (0.74–11.62)	2	*	**5**	**4.81 (1.31–17.63)**	0.17
*Diabetic population*										
Metformin use (years)**	43	35	0.95 (0.86–1.04)	24	**0.89 (0.81–0.99)**	3	1.11 (0.95–1.28)	8	1.02 (0.92–1.13)	**0.01**
Sulfonylurea use (years)**	24	18	1.05 (0.99–1.13)	13	1.03 (0.93–1.13)	1	*	4	**1.10 (1.00–1.20)**	0.40
Insulin use (years)**	6	20	1.10 (0.98–1.23)	13	1.11 (0.93–1.33)	2	*	5	1.07 (0.83–1.38)	0.91

Numbers may differ due to lack of information on tumor receptors in some participants

Models for diabetes and diabetes management are adjusted for age, study level (no studies/<high school/high school/>high school), BMI (continuous), age at menarche, age at first birth, existence of previous biopsies and family history of the studied cancer (none/one first degree/more than one first degree/second degree)

Models for metformin and sulfonylurea further adjusted for insulin treatment (yes/no) and for treatment with other hypoglycemic agent (yes/no), while models for insulin are adjusted for treatment with metformin (yes/no) or sulfonylurea (yes/no)

Associations that reach statistical significance are in bold

* Values are not presented due to the small number of cases in these subgroups

** Based on diabetic participants who had received drug treatment for at least 1 year

The last part of Table 2 shows the association between duration of specific anti-diabetic treatments and breast cancer risk considering only diabetic women. Analyses based on metformin use alone showed a heterogeneous effect of this drug over different tumor subtypes: While the number of years under metformin treatment was not associated with the risk of HER2 or TN tumors, a negative association with the risk of HR+/HER2− breast cancer was observed (OR_per year_ 0.89; 95 % CI 0.81–0.99). Duration of treatment with sulfonylurea was associated with a nonsignificant increased risk of breast cancer, after adjustment for insulin and metformin use (OR_per year_ 1.05; 95 % CI 0.99–1.13). Although we could not evaluate the dose–response association between years of insulin glargine use and cancer risk due to a lack of power, we found that women who were receiving insulin glargine at the time of the interview had an increased risk of breast cancer when compared to women who had never received this drug (OR 4.97; 95 % CI 1.09–22.7).

Interaction analyses revealed no effect modification of BMI on the association between diabetes or diabetes management and breast cancer risk.

## Discussion

Our findings do not support an overall association between diabetes and breast cancer, although they suggest an increased risk of TN tumors in postmenopausal diabetic women. They also suggest that insulin use may increase the risk of breast cancer. Finally, results among diabetic patients indicate that the risk of developing HR+/HER2− tumors may decrease with longer metformin use, while the risk of TN tumors may increase with a longer duration of sulfonylurea use.

Despite a number of meta-analyses linking diabetes and breast cancer incidence in postmenopausal women, results from three recently published large population-based studies have raised uncertainty. The first of these null studies retrospectively evaluated the risk of breast cancer in postmenopausal women using data from the Columbia Linked Health Database [8]. The second, based on record linkage between the Danish National Diabetes Register and Cancer Registry, found no association between diabetes prevalence or diabetes duration and breast cancer risk [9]. The third, which included 68,019 postmenopausal women followed over a mean of 11.8 years, also failed to find an overall increased risk of breast cancer among those with diabetes [10]. However, results from this last study were modified when diabetes medication was taken into account, as a reflection of the importance of considering diabetes treatment when studying the risk of cancer associated with this disease. Women under treatment with “drugs other that metformin” showed a nonsignificant increased risk of breast cancer (hazard ratio 1.16; 95 % CI 0.93–1.45), while those receiving metformin presented lower incidence (hazard ratio 0.75; 95 % CI 0.57–0.99).

Differences in breast cancer risk factors by intrinsic subtypes are well documented, and some authors have suggested that TN tumors may be more strongly associated with insulin resistance [11]. Our results, showing a strong association between diabetes and incidence of TN tumors, would support this hypothesis. However, the few previous epidemiological studies that have evaluated the influence of diabetes on different breast cancer subtypes have yielded contradictory results. In the Carolina Breast Cancer Study, an increased risk of basal-like breast tumors was seen in postmenopausal women with higher waist circumference and waist-to-hip ratio [12]. However, although these measures of central adiposity are well-known markers of insulin resistance and were strongly associated with a history of diabetes, no elevated prevalence of diabetes was found in women with TN tumors when compared to other breast cancer subtypes [12]. Results from the Study of Osteoporotic Fractures showed that diabetes was associated with a borderline significant increased risk of ER+ and PR+ cancers, while no effect was seen for ER− or PR− [7]. Finally, a retrospective cohort study focusing on the metabolic syndrome as a whole instead of diabetes as an individual component found a higher prevalence of this syndrome in patients with TN tumors, with blood glucose being an independent risk factor for this specific subtype [13]. In light of these conflicting results, additional research is warranted.

The biological mechanisms behind a potential increased risk of breast cancer in women with diabetes are unknown, although are probably related to alterations in circulating concentrations of insulin, insulin-like growth factors and endogenous sex hormones. Insulin and IGF-1 receptors are frequently expressed in breast cancer cells [14], with evidence that their signaling pathways are of crucial importance in the role of breast cancer tumorigenesis [15, 16], also in the case of TN tumors [17]. Insulin has a paracrine effect on secretion of adipokines [18], which may contribute to the increasing risk of more aggressive breast tumors. Additionally, insulin can inhibit the production of sex hormone-binding globulin in the liver [19], with subsequent increased levels of free estradiol and increasing proliferation of breast epithelial cells.

### Diabetes treatment and risk of breast cancer

#### Metformin

Metformin is the most commonly used drug in patients with type 2 diabetes. Experimental studies have shown that this biguanide drug is capable of inhibiting the proliferation of breast cells [20]. Additionally, it can induce apoptosis of TN [21] and HER2-positive cells [22], and it can repress the process of epithelial-to-mesenchymal transition [23]. Interestingly, metformin also reduces the growth of several tumoral xenografts in mice including those established from breast cancer cells [24].

The evidence on the association between metformin use and the risk of breast cancer has been mixed in epidemiologic studies [25–27]. Results from most meta-analyses have suggested a nonsignificant decreased risk of breast cancer in metformin users [25, 26], while no effect has emerged from randomized clinical trials (RCTs) [26]. To date, only one previous study, based on data from the Women’s Health Initiative clinical trials, has evaluated the incidence of specific subtypes of breast cancer in diabetic women under metformin treatment [10]. Interestingly, findings from this study were very similar as those from our study, with a protective effect of metformin only observed for ER+/PR+ and not ER−/PR− tumors.

#### Sulfonylurea

A meta-analysis using data from 296,904 subjects in cohort studies found an increased risk of total cancer among participants using sulfonylureas when compared to non-sulfonylurea users [28]. However, data from 6573 subjects in RCTs and 12,040 subjects in case–control studies failed to demonstrate a similar association [28]. Regarding breast cancer, the evidence is scarce and the few existing studies have yielded inconsistent findings [29–31]. Our results point toward a possible detrimental effect of sulfonylureas on the risk of TN breast cancer.

#### Insulin

Insulin is a well-known growth factor with particularly strong mitogenic effects over cancer cells. According to our results, showing an increased prevalence of insulin use among cases than among controls, insulin treatment may increase the risk of breast cancer. However, because longer duration of insulin use among diabetics was not associated with breast cancer risk, this positive association cannot be easily explained based on the mitogenic effects of this drug and it can also reflect the influence of a longer duration of diabetes among insulin users.

Results from a meta-analysis of observational studies have lately shown an overall increased risk of cancer in patients treated with insulin, although a nonsignificant increased risk of breast cancer (RR 1.86; 95 % CI 0.92–2.98) was reported [32]. Data from RCTs do not support that insulin increases the risk of total cancer, although interpretation of the results is limited because cancer is not among the end points of interest. Additionally, the few RCTs on insulin therapy that have reported data on cancer have focused on mortality [33].

Available data from in vitro experiments suggest that insulin glargine may have greater proliferative effects than human insulin in some breast cancer cell lines [34]. This finding is supported by observational studies showing that glargine use may be associated with an increased risk of breast cancer [35], at least at high doses and with long duration of treatments [5, 33]. A population-based cohort with more than 27,000 users of insulin glargine and 100,757 users of NPH has shown a 30 % increased risk of breast cancer in patients with ever use of glargine (RR 1.3; 95 % CI 1.0–1.8) [36]. Our results also suggest an increased risk of breast cancer, although the number of women that specifically reported the use of glargine was too small to reach any firm conclusion.

### Strengths and limitations

This is the first population-based study that evaluates the association between diabetes and breast cancer in the Spanish population. Our main strength is that we have histologically confirmed incident cases and that we have classified tumors according to their receptor status. Very few studies have previously evaluated the association between diabetes and breast cancer by specific subtypes, and our results suggest the importance of this approach.

Since our results are based on a case–control study, several methodological limitations may exist. First, diabetes history is self-reported and so it is subjected to recall bias. According to the International Diabetes Federation, around 35 % of the European diabetic population is unaware of having this condition [1]. However, we expect underdiagnosis not to be so important in our population because participants had frequent contact with the health system, as reflected by their high prevalence of screening behaviors. Additionally, results from a meta-analysis on the association between diabetes and breast cancer showed that findings were unchanged when the diagnosis of diabetes was self-reported or confirmed with medical records [37]. Second, we were limited by the sample size, particularly for evaluating certain subgroup associations. Third, it is hard to disentangle the effects of diabetes treatment from those of the disease itself. As an example, diabetic patients receiving insulin are a subgroup with very specific characteristics; they usually have more severe forms of diabetes and greater prevalence of comorbidities that can lead to an increased risk of cancer. Fourth, individuals receiving insulin usually visit their doctors more frequently, and this may increase their probability of screening and cancer detection. Additionally, we do not have information on glycemic control, and we can therefore not evaluate its potential mediating role in the observed associations. Finally, although many confounders have been taken into account, we cannot rule out some residual confounding by lifestyle-related factors associated with both diabetes and breast cancer development.

## Conclusions

Our findings do not support an overall association between diabetes and breast cancer, but they provide some suggestive hypothesis for associations with particular tumor subtypes. Although our results should be interpreted with caution due to limitations in sample size, results suggest that diabetes may increase the risk of TN breast tumors. Given the low survival rates in women diagnosed with TN breast tumors, more studies are needed to better characterize this association.
